# Successful Laparoscopic Management of Ruptured Tubal Pregnancy with an Ipsilateral Ectopic Pelvic Kidney

**DOI:** 10.1155/2014/682737

**Published:** 2014-07-21

**Authors:** Jimmy Belotte, Jim Belotte, Mitchell Alexis, Awoniyi O. Awonuga, Tina Jessica Aguin

**Affiliations:** ^1^Department of Obstetrics and Gynecology, C. S. Mott Center for Human Growth and Development, Wayne State University School of Medicine, Wayne State University, 275 E. Hancock, Detroit, MI 48201, USA; ^2^Department of Surgery, Nassau University Medical Center, 2201 Hempstead Turnpike, East Meadow, NY 11554, USA; ^3^Department of Obstetrics and Gynecology, Wayne State University School of Medicine, Wayne State University, 275 E. Hancock, Detroit, MI 48201, USA

## Abstract

*Objective.* To report a case of successful laparoscopic management of a left ruptured tubal pregnancy in the setting of an ipsilateral ectopic pelvic kidney. *Method.* Case report was prepared at Wayne State University/Detroit Medical Center. The patient is a young woman gravida 2 para 0 in her twenties who presented with severe abdominal pain and vaginal bleeding. She had a plateaued beta HCG and ultrasonographic findings suggestive of ectopic left tubal pregnancy along with an ectopic ipsilateral pelvic kidney. The IRB approval is not needed, as this is a case report. The informed consent could not be obtained, as the patient was not reachable. *Result.* Multiple intraperitoneal adhesions, left ruptured ampullary ectopic pregnancy and left retroperitoneal pelvic mass consistent with ipsilateral ectopic pelvic kidney. *Conclusion.* Laparoscopic management of tubal pregnancy can be safely performed in the setting of an ipsilateral ectopic pelvic kidney.

## 1. Background

In the United States, the rate of ectopic pregnancy (EP) has increased by almost fourfold, from 4.5 to 16.0 per 1,000 reported pregnancies, from 1970 to 1989. In women suspected to have an EP, serial beta HCG measurements and transvaginal ultrasound (TVS) evaluation remain the mainstay of the diagnosis. Aside from the diagnosis of EP, TVS evaluation of the pelvis can allude to the presence of other pelvic pathologies, such as pelvic masses, that may modify the management or surgical approach to treatment. Although not often appreciated, congenital anomalies of the genitourinary system such as a pelvic kidney can present as a pelvic mass on bimanual examination of the pelvis and are confirmed by imaging such as TVS during evaluation of a patient suspected to have an EP. Congenital anomalies of the kidney and urinary tract (CAKUT) represent a broad range of disorders with an incidence of 1 in 1130 [1]. Moreover, CAKUT can accompany congenital anomalies of the Mullerian duct system and patients with the latter are at increased risk of an EP [2, 3]. When medical treatment is not indicated, operative laparoscopy is the most common surgical approach because of its established superiority [4].

Here, we present a case of successful laparoscopic management of a left ruptured tubal pregnancy in the setting of an ipsilateral ectopic pelvic kidney.

## 2. Case Report

**Figure 1:**
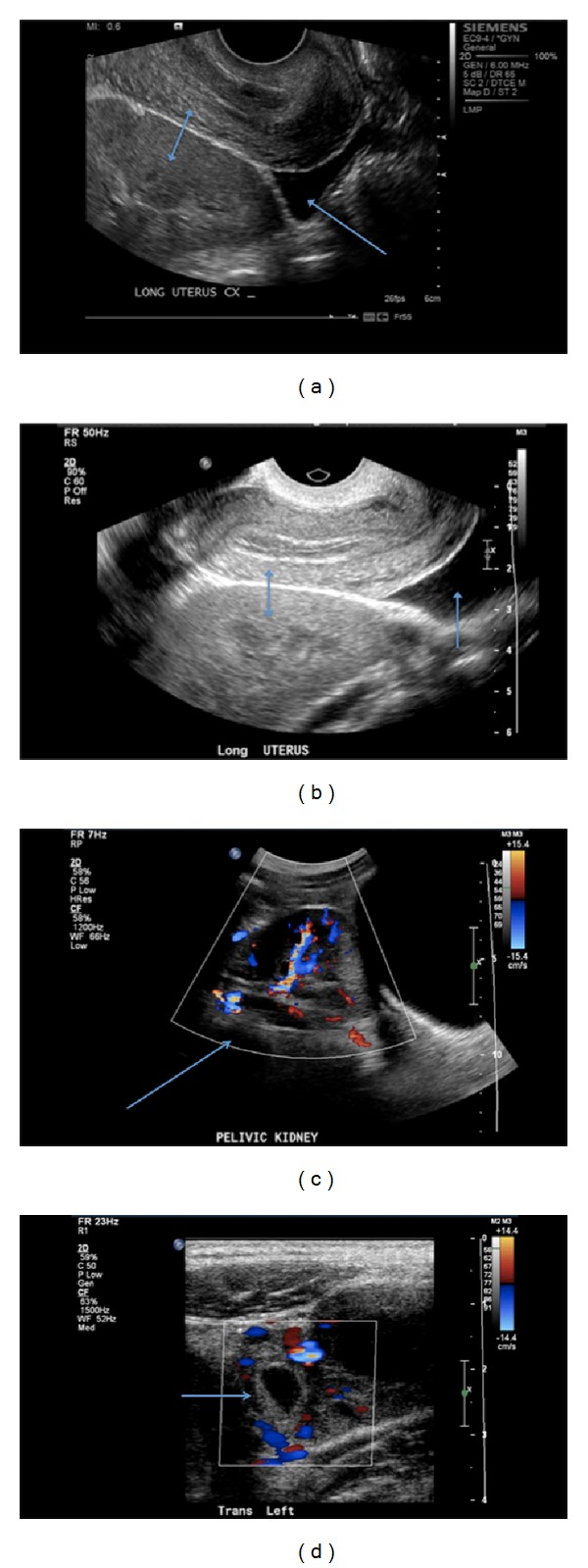
(a) Longitudinal view: an empty uterus superiorly and pelvic kidney inferiorly (double arrow), free fluid in the cul-de-sac (single arrow). (b) Longitudinal view: an empty uterus superiorly and pelvic kidney inferiorly (double arrow), free fluid in the cul-de-sac (single arrow). (c) Longitudinal view: ectopically located pelvic kidney with color Doppler (single arrow). (d) Transverse view: hypoechoic cystic structure with vascular flow in the left adnexa depicting a tubal pregnancy (single arrow).

A woman G2P0010 in her twenties presented to the emergency room (ER) with a two-day history of nausea and vomiting, severe abdominal pain, vaginal spotting, and a missed menstrual period. Her symptoms began with vaginal spotting two days earlier and were followed with abdominal pain. She reported that the abdominal pain has improved from 10 out of 10 to 2 out of 10 in severity on the visual analog pain scale within the last 24 hours. Besides being diagnosed and adequately treated for genital chlamydia infection in 2006, her past histories are unremarkable. She is a social drinker, smoked cigarettes at a frequency of one pack per day for the last 5 years and also smoked marijuana. On physical examination, she was in no apparent distress with normal vital signs. A mild tenderness to palpation was elicited in the lower abdomen without rebound. Pelvic examination demonstrated a normal 6-week size anteverted but nontender uterus and mild-to-moderately tender left adnexa. The complete blood count was normal. She had a positive urine pregnancy test and a quantitative beta HCG of 1243 mIU/mL. A transvaginal ultrasonography (TVS) showed an empty uterus, a small amount of free fluid in the cul-de-sac, and an incidental left pelvic kidney (Figures 1(a), 1(b), and 1(c)). The patient was deemed stable with minimal pain and therefore was discharged home with instructions to follow up within 48 hours. Thirty-six hours later, she presented to our Institution with similar symptoms. At that time, the beta HCG increased 2673 mIU/mL and a repeat TVS revealed the hitherto diagnosed pelvic kidney, a 3-4 cm mass (Figure 1(d)) in the left adnexa, and an empty uterus with small amount of free fluid in the pelvis.

**Figure 2:**
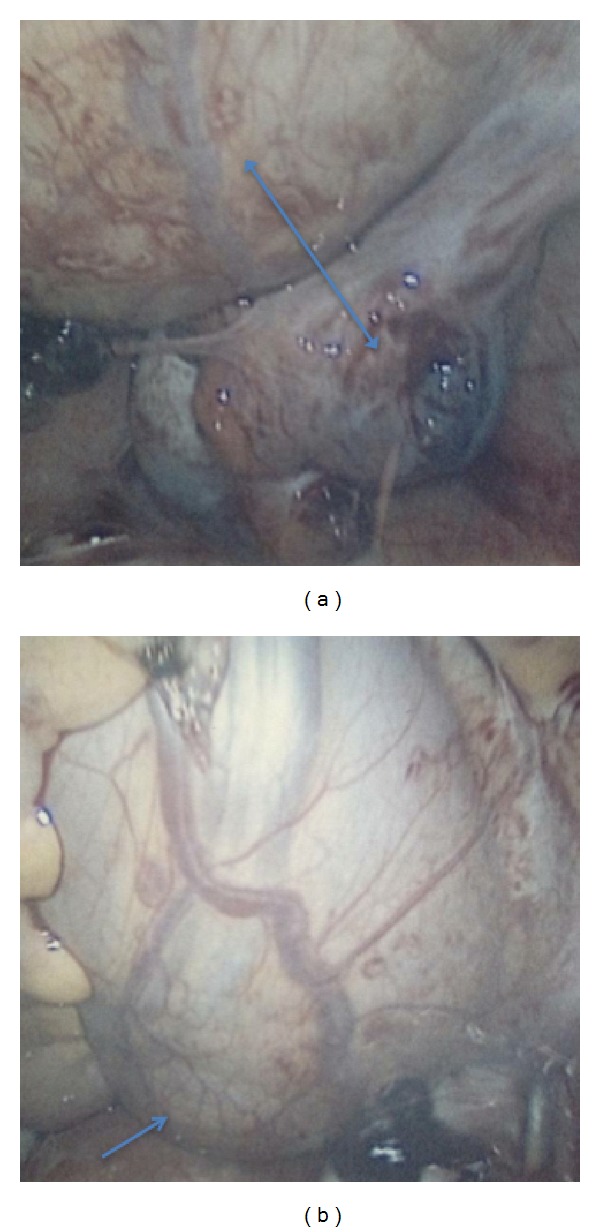
Laparoscopy: (a) pelvic kidney superiorly with a ruptured tubal ectopic pregnancy inferiorly (double arrow) and (b) ectopic pelvic kidney.

After discussing the risks, benefits, indications, and alternatives to surgical intervention, she opted for and consented to laparoscopic surgery with the understanding that the surgery may be converted to laparotomy. Mindful of the presence of the pelvic kidney, accessory port placements were modified. Abdominal gas insufflation and umbilical port placement were performed using the Veress needle and optical trocar, respectively. The accessory trocars were placed under direct visualization as follows: a 5 mm trocar suprapubically in the midline and a 10 mm trocar on the right 12 cm lateral to the midline and 8 cm superior to the right anterior superior iliac spine for the specimen removal. Upon entry, no injuries were appreciated and multiple adhesions were noted specifically, between the omentum, the anterior abdominal wall, and the left pelvic sidewall. A 12 cm bulge (Figure 2(a)) was observed on the left at the level of the pelvic brim retroperitoneally, which was consistent with an ectopic pelvic kidney. Also, a 3 cm ruptured left ampullary mass, consistent with an EP, was visualized in a distorted left fallopian tube (Figure 2(b)). The adhesions were lysed and a left salpingectomy was performed without complications. After a brief stay in the Post Anesthetic Care Unit (PACU), the patient was discharged home in stable condition. She was subsequently seen in the office two weeks later and had an uneventful postoperative recovery. Histopathology report of the excised fallopian tube confirmed the ruptured tubal pregnancy with features of chronic salpingitis.

## 3. Discussions

Worldwide, the complication rate related to laparoscopy in gynecology is estimated to be between 0.2 and 10.3% with operative laparoscopy rate as high as 18% [5]. Trocar and Veress needle injuries account for the majority of avoidable serious complications at entry. The mortality rate from those complications averages 5% and most commonly involves injuries to the common iliac vessels, aorta, and inferior vena cava [6]. Concomitant presence of pelvic adhesions and other pelvic masses distort the pelvic anatomy and therefore can predispose the bowel or other pelvic structures to injury during laparoscopic surgery. Foreknowledge of the presence of pelvic masses is increasingly possible with the advent of ultrasound evaluation of patients with pelvic pain and suspected EP. Every effort should be made to rule them out before the intended surgery [7]. One serious consideration in the preoperative evaluation was the risk/benefit profile of laparoscopic management versus minilaparotomy. In this case, we took a methodical and multidisciplinary approach in our assessment; first we reviewed the ultrasonographic findings with the radiologist with particular attention to the size, location, and Doppler studies of the ectopically located kidney. Second, we discussed the feasibility of safe operative laparoscopy such as port placement and methods with emphasis on open (Hasson) versus closed (Veress needle) approaches, as well as Palmer's point versus supraumbilical primary port placement. Third, all options were presented to the patient with pros and cons of each approach. Mindful of the presence of the pelvic kidney, we modified our surgical approach to avoid injury to the pelvic kidney. Instead of using routine bilateral lower quadrants sites for accessory ports, we opted for a right lower quadrant and a suprapubic trocar sites with placement under direct visualization. As mentioned above, the surgery was uneventful, with minimal blood loss, short operative time, and fast recovery, all the benefits of minimally invasive surgery. Besides the risks inherent to general anesthesia and routine laparoscopic salpingectomy, the patient was not subjected to any additional and unnecessary risks.

The challenges posed by a pelvic kidney in the setting of an ectopic pregnancy have been previously described in the literature and they involve not only those with undiagnosed congenital anomalies, but also those with a kidney transplant. In one report, accidental puncture of a pelvic kidney complicated culdocentesis which is an obsolete diagnostic approach for ectopic gestation, at least in the Western world [8]. This case reinforces the need for proper preoperative assessment before laparoscopic surgery even in emergencies. In this report, we hope to increase awareness of gynecological surgeons to the need for proper preoperative evaluation by ultrasound to exclude other pelvic masses, including ectopic pelvic kidney so that significant morbidity can be avoided by modification of entry ports during operative laparoscopy. Additionally, this case demonstrates that laparoscopic salpingectomy for ruptured ectopic pregnancy can be safely performed in the presence of an ectopically located pelvic kidney on the same side.
